# ChatGPT for Improving Medical Education: Proceed With Caution

**DOI:** 10.1016/j.mcpdig.2023.04.006

**Published:** 2023-06-30

**Authors:** Thomas Woodland

**Affiliations:** a Department of Anaesthetics, Bristol Royal Infirmary, Bristol, United Kingdom

The public release of ChatGPT^1^ and Google Bard^2^ has generated considerable interest in the rapidly advancing capabilities of large language models (LLMs), a form of artificial intelligence (AI). Large language models are neural networks trained on billions of words of text, giving them the ability to recognize patterns and associations within the language. This information allows the models to make predictions about which words or phrases will follow, resulting in the generation of natural-sounding prose. A notable strength of these models is their capacity to process dense medical text into concise and comprehensible summaries.^3^ At times, it is easy to forget that you are conversing with a computer because it responds to seemingly any question or prompt using fluent and coherent language.

The impact on education is likely to be substantial, offering the potential to develop critical thinking abilities, summarize and explain complex concepts, and aid teachers.^4^ However, LLMs are an emergent technology, still within the testing phase of their first public release. Learners seeking ways to further their own educational needs must be aware of the current limitations and potential benefits.

Traditional search engines, such as Google, work by systematically visiting web pages and archiving their data in an indexed database. On receiving a user’s search query, the engine determines the results to be displayed based on an evaluation of several parameters, including quality and relevance of the content, and the degree of user engagement. This means that although they may be excellent at returning websites with content that precisely matches the user’s search query, they fall short when trying to answer more abstract prompts that require evaluation of semantics and reasoning.^5^ For example, a query submitted through a conventional search engine containing a diverse range of terms may return results that contain some of the keywords but, individually, have little relation to the search query as a whole. In contrast, an LLM can perform sentiment analysis on the query, determine exactly what the user is trying to ask, extract information from a range of sources, and synthesize a response. The ability of LLMs to answer complex questions in this manner could help students form an understanding that bridges the divide between didactic resources, such as textbooks, and the nuances of the clinical environment.

In part, this is because communication with LLMs follows a natural conversational pattern, resembling how the learner might interact with a teacher. Rather than passively reading information from a textbook, students can actively engage with the content provided. The learner can further their comprehension by querying the information provided, asking for clarifications, and exploring how concepts are interrelated. Active engagement promotes explorative and curious learning, which is essential in helping convert surface-level knowledge to a deeper understanding.^6^

Given the marked capabilities, it can be easy to forget that one is conversing with an AI model. The text generated is fluent, literate, and convincing. The knowledge base that the model draws from is vast, and it appears to be able to answer any question with an intelligent and considered reply. However, learners must understand that in their current form, LLMs are far from infallible. Despite initial appearances, they lack understanding of the meaning or broader context of the sentences they generate. Couple this with the appreciation that LLMs have no perception of how the world functions and their limitations start to become apparent.

The tendency of AI to produce false or misleading outputs is termed as AI hallucination, which occurs when the generated text is not based on a factual source but results from statistical predictions of what words are likely to follow the given input.^7^ Of particular concern is their propensity to produce references that appear genuine but, on closer inspection, are revealed to be entirely fabricated.^8^ A thorough understanding of the subject matter is necessary to discern and refute incorrect answers produced by the model. Although this may be trivial for subject experts, it markedly limits the usefulness of LLMs for learners trying to develop their own understanding.

Because of the conversational nature of these interactions, it is easy to subconsciously anthropomorphize the model and attribute it with human-level cognitive abilities. It is important to remember that LLMs learn statistical patterns and word associations; they do not truly understand the meaning or implications of the language they generate.^9^ LLMs do not possess innate knowledge of how the world functions or the cognitive processes that underpin human existence, nor do they have any notion of truth or falsehood, only the statistical likelihood of semantics,^10^ and these models are not explicitly programmed to provide truthful responses.

Judicious use of this technology demands appreciation of its capabilities and limitations. Medical practice requires the ability to analyze and evaluate information, interpreting each result within the specific clinical context. Given the limitations inherent in current models, using an LLM to inform medical learning without subjecting its output to a high degree of critical evaluation could be unethical and potentially harmful. As the technology evolves, learners must maintain an informed and discerning stance in appraising the responses generated. Large language models are not a substitute for medical knowledge or understanding but a tool that can complement and augment them.

## Potential Competing Interests

The author reports no competing interests.
